# Quantitative Assessment of Tissue Biomarkers and Construction of a Model to Predict Outcome in Breast Cancer Using Multiple Imputation

**DOI:** 10.4137/cin.s911

**Published:** 2008-12-23

**Authors:** John W. Emerson, Marisa Dolled-Filhart, Lyndsay Harris, David L. Rimm, David P. Tuck

**Affiliations:** 1 Department of Statistics, Yale University, New Haven, Connecticut 06520; 2 Department of Pathology, Yale University School of Medicine, New Haven, Connecticut 06510; 3 Medical Oncology, Yale University School of Medicine, New Haven, Connecticut 06510

**Keywords:** biomarker, immunohistochemistry, multiple imputation, variable selection, breast cancer

## Abstract

Missing data pose one of the greatest challenges in the rigorous evaluation of biomarkers. The limited availability of specimens with complete clinical annotation and quality biomaterial often leads to underpowered studies. Tissue microarray studies, for example, may be further handicapped by the loss of data points because of unevaluable staining, core loss, or the lack of tumor in the histospot. This paper presents a novel approach to these common problems in the context of a tissue protein biomarker analysis in a cohort of patients with breast cancer. Our analysis develops techniques based on multiple imputation to address the missing value problem. We first select markers using a training cohort, identifying a small subset of protein expression levels that are most useful in predicting patient survival. The best model is obtained by including both protein markers (including COX6C, GATA3, NAT1, and ESR1) and lymph node status. The use of either lymph node status or the four protein expression levels provides similar improvements in goodness-of-fit, with both significantly better than a baseline clinical model. Using the same multiple imputation strategy, we then validate the results out-of-sample on a larger independent cohort. Our approach of integrating multiple imputation with each stage of the analysis serves as an example that may be replicated or adapted in future studies with missing values.

## Introduction

When confronted with missing data, investigators often choose to drop cases with missing values from their analysis (*case deletion*). Sometimes, this is a conscious decision, while at others it is a side-effect of statistical software. Occasionally, missing values are “filled in,” perhaps by inserting the mean or median of the non-missing values. The term *imputation* is used to describe the general act of filling in missing data, and many approaches to imputation have been proposed and studied. The evolution of the EM algorithm (Dempster, Laird, and Rubin, 1977) and techniques related to Markov chain Monte Carlo (MCMC) led to the development of *multiple imputation* (see Rubin, 1987, 1996; and Schafer, 1997, for example). As implied by the name, the imputation of missing values is conducted multiple times, leading to multiple realizations of complete data sets. The statistical analysis is conducted on each imputed data set in turn, and the results are pooled. When values are *missing at random* (see Rubin, 1976), multiple imputation can lead to more efficient, statistically valid inferences than case deletion or other methods of imputation. A full discussion of the field is beyond the scope of this paper, and we refer interested readers to approachable references such as Schafer (1999). This paper presents the analysis of the prognostic value of protein biomarkers measured on a tissue microarray from a hospital-based cohort of breast cancer patients from Yale. In this study, about 20% of the expression levels were missing because of unevaluable staining, core loss, or lack of tumor in the histospot. We developed a technique based on multiple imputation to address the missing value problem, first selecting markers using a training cohort and later validating the results out-of-sample on a validation cohort.

Breast cancer is increasingly recognized as a disease marked by heterogeneous cellular behavior and response to anticancer therapies. Different, highly targeted anticancer therapeutic agents play an increasing role in cancer treatment, driving the need to identify molecularly defined subtypes of breast cancer as well as predictive and prognostic biomarkers for characterizing particular subsets of patients. The rise in popularity of molecularly defined diagnostic tests, such as the OncotypeDx test in breast cancer (Paik, 2004), illustrates the desire of patients and oncologists to customize therapy using information about the biological features of tumors. However, this type of analysis depends upon mRNA expression from the primary tumor and does not reflect the most relevant targets: proteins which are misregulated within the cancer cell. Furthermore, RNA has less stability than proteins and is more difficult to preserve. Although protein expression levels are routinely assessed using immunohistochemistry in determining therapeutic regimen in breast cancer patients, their usage, historically, has been limited by non-quantitative methods.

The recent development of AQUA™ algorithms allows quantitative assessment of protein expression levels within specific subcellular compartments. A series of images are collected by a custom microscope platform. The amount of protein expressed within the compartment is then quantified by co-localization using molecular methods to define subcellular compartments. This methodology, including details of the out-of-focus light subtraction imaging methods required is described in Camp (2002).

Several breast cancer studies (Zhang, 2003; Makretsov, 2003; Korsching, 2002; Arnes, 2005; Foulkes, 2004; Foulkes, 2003; Nielsen, 2004; van de Rijn, 2002; Makretsov, 2004; Jacquemier, 2005) have developed models for predicting survival using positive/negative immunohistochemistry (IHC) scoring. Here, we use AQUA^™^ to develop a prognostic model of patient outcomes based on protein expression levels that is as effective as a model utilizing the nodal status determined by an axillary lymph node biopsy. Specifically, we use a training cohort of 236 patients to identify a small subset of protein markers important in predicting patient survival. We then use an independent validation cohort of 338 patients to examine the relative prognostic value models containing these markers and/or nodal status. Each step of the analysis depends on multiple imputation to address the missing value problem. We found that the addition of either nodal status or the four protein expression levels provides similar (and significant) improvement over a baseline model consisting of age, tumor size, and nuclear grade. However the best models combine the nodal status with the protein expression data.

This paper is able to confirm the importance of several protein markers known from previous studies and points to the possible importance additional markers. Second, we introduce a strategy for statistical analysis in the presence of missing measurements, and hope this will provide basis for similar analyses in the future. A simple simulation demonstrates the advantage of the procedure over several alternatives. We recommend both the use of multiple imputation and out-of-sample validation of results.

## Methods

### Patient cohorts

The full breast cancer cohort consists of 677 samples of archived invasive ductal carcinoma tissue which have been described in previous studies (Dolled-Filhart, 2006). The training set of 250 cases (McCabe, 2005) was selected from the full cohort taking into account tumor availability and designed to contain half node-positive and half node-negative specimens; 14 of these cases were excluded because of insufficient breast tumor epithelium or lack of clinical follow-up. The remaining 427 cases comprised the validation set; 89 of these cases were excluded because of either insufficient breast tumor epithelium or lack of clinical followup. Thus, the final training and validation cohorts contain 236 and 338 cases, respectively. Yale University Human Investigation Protocol 8219 approved all personal health information collection by informed consent signed at the time of surgery.

### Tissue microarray construction

Details of the tissue microarray construction are available in previously published studies (Camp, 2003; Dolled-Filhart, 2005; Dolled-Filhart, 2004; McCabe, 2005). We used formalin-fixed paraffin-embedded breast cancer tumors from the archives of the Yale University from 1961 to 1983 with approximately half node-positive specimens and half node-negative specimens, as assessed in other studies (Dolled-Filhart, 2003; Kang, 2003; Ocal, 2003; Chung, 2004; Kluger, 2004; Camp, 2003; McCarthy, 2005). The regions of invasive ductal carcinoma were selected by pathologists, and cores were 0.6mm in diameter. We used a manual Tissue Microarrayer (Beecher Instruments, Silver Spring, MD), and adhered 5um sections of the tissue microarrays to slides using an adhesive tape-transfer method (Instrumedics, Inc., Hackensack, NJ) and UV crosslinking.

The published results of breast cancer RNA expression profiling studies were used to construct a list of candidate biomarkers for screening by AQUA™ analysis of breast cancer tissue microarrays. The list was narrowed to those with available antibodies with previous western blotting or immunohistochemical validation in the literature. Table 1 presents the 42 markers and antibodies used in studying the training cohort; a subset of markers was assessed in the validation cohort.

### Immunofluorescence staining and image analysis

The tissue microarrays were deparaffinized by two thirty-minute xylene rinses, two one-minute 100% ethanol rinses, and a one-minute rinse in water. The slides were pressure cooked in a sodium citrate buffer (pH 6.0) to allow antigen retrieval. Following a brief rinse in 1×Tris-buffered saline (1×TBS), endogenous peroxidases were blocked with a thirty minute 2.5% hydrogen peroxide/methanol incubation. The one hour incubation with 0.3% bovine serum albumen (BSA) reduced nonspecific background staining. Tissue microarrays were incubated overnight at 4 °C with anti-cytokeratin antibody (monoclonal anti-cytokeratin clone AE1/AE3 or rabbit anti-cytokeratin wide spectrum, DAKO, Carpinteria, CA, 1:100) and the target antibody for each slide. The dilutions, incubation times, and sources of each of the 43 antibodies utilized in this study are included in Table 1. Following three washes of five minutes each in 1×TBS, 1×TBS/Tween and 1×TBS, slides were incubated with secondary antibodies: cytokeratin detection (Alexa 488 goat anti-mouse or Alexa 488 goat anti-rabbit, 1:100, Molecular Probes, Eugene, OR), DAPI (6-diamidino-2-phenylindole, 1:100, DAKO) and species specific horseradish peroxidase (HRP) with a dextran-polymer backbone (Envision, DAKO) for the rabbit and mouse target antibodies. Goat primary target antibodies were incubated with biotinylated anti-goat (1:200, Vector, Burlingame, CA) and Cy-2-donkey anti-mouse to detect cytokeratin (1:50, Jackson Laboratories, Bar Harbor, Maine), followed by TBS washes and incubation with Streptavidin HRP (1:200, Perkin Elmer) and DAPI (6-diamidino-2-phenylindole, 1:100, DAKO) for one hour. Following the TBS washes, all slides were incubated for ten minutes with Cy-5 tyramide for all target antibodies (1:50 dilution in Amplification Diluent, Perkin Elmer) because its emission spectra are outside the tissue autofluorescence spectra. The slides were mounted in 0.6% n-propyl gallate (an anti-fade mounting medium) and coverslipped.

AQUA™ software linked to an Olympus AX-51 epifluorescence microscope provided measurements of the proteins within the epithelial regions of each tissue microarray core (as previously described in Camp (2002). High resolution monochromatic images of each histospot (1024 × 1024 pixels, 0.5 um resolution) were captured for each fluorescent signal (DAPI staining to identify nuclei, Alexa488 for cytokeratin, and Cy5 for target antibodies). AQUA™ analysis separated epithelial cells from stromal regions based on cytokeratin expression. Similarly, nuclear regions were identified using DAPI positivity. The target pixel intensity is divided by the total area of epithelial regions (or DAPI positive regions for nuclear expression) to generate an AQUA™ score normalized for differences in microarray core epithelial area.

### Statistical analysis: overview

All analyses were conducted in the R statistical programming environment (R Development Core Team, 2007, http://www.R-project.org), and baseline clinical variables (age at diagnosis, tumor size, and nuclear grade) were included in every model.

Forty-two proteins were evaluated in the training cohort. Initial analysis of the training cohort identified 15 of the most promising markers (marker selection). At the same time, a “best” model was identified (model selection), consisting of 4 of these 15 markers. The results were validated using the validation cohort (out-of-sample validation), providing an objective and rigorous means of evaluating the candidate model. Measurements on the validation cohort were obtained only for the 15 identified markers, and were used to compare the prognostic value of several models (described below). All measurements were log transformed and normalized to have zero mean and unit variance, and we used Cox proportional hazards models of patient survival time. Approximately 20% of the tissue microarray measurements were missing because of core loss, unevaluable staining or the lack of tumor in the histospot. As a result, the marker and model-selection methodologies as well as the out-of-sample validation utilized multiple imputation techniques.

The most common approach to dealing with only a few missing values—casewise deletion—is impractical with many missing values. The alternative is *imputation*, the general act of filling in missing data, and many approaches to imputation have been proposed and studied (see Schafer for, 1999 an overview). Investigators may sometimes fill in missing values using the mean or the median of each variable; a more advanced approach would rely on the *k*-nearest neighbor algorithm (filling in missing values based on the mean or median of the *k* nearest neighbors as identified by non-missing variables). Neither of these approaches is ideal, because the subsequent analyses fail to account for the uncertainty due to the missing values, but multiple imputation specifically addresses this issue. The imputation of missing values is conducted multiple times, leading to multiple realizations of complete data sets, the statistical analysis is conducted on each imputed data set in turn, and the results are pooled. When values are *missing at random*, multiple imputation can lead to more efficient, statistically valid inferences than case deletion or other methods of imputation.

Our multiple imputation procedure is one of the simplest, based on the multivariate normal distribution. With *p* variables, however, it requires estimation of *p* + *p*(*p* + 1)/2 parameters (*p* means and a covariance matrix); this would be infeasible with the original 42 markers in a study of this size (a training cohort of approximately 1500 would be needed). As a result, we limited its use to no more than a subset of 9 variables at a time from the training set. This choice is based on the size of this particular study; a different choice would be needed for other studies. We used the multiple imputation tools in the “norm” package (Novo 2002) in the R Statistical software, to obtain random draws from the incomplete multivariate normal distribution of missing data conditional on the non-missing values. Each application of multiple imputation involves the analysis of many different complete data sets (differing in the imputed values), leading to many slightly different analyses. The results of these analyses are then combined, providing a natural way of incorporating the uncertainty due to the missing values.

### Statistical analysis: marker selection

**Figure 1:**
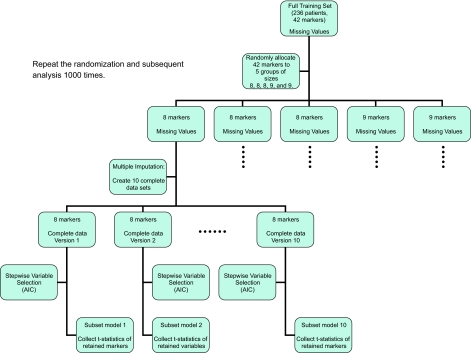
Marker Selection.

In the first phase of the study, we chose 15 of the most promising markers for subsequent measurement on the validation cohort. This number was chosen partly in recognition of the anticipated use of multiple imputation with 338 cases in the validation cohort, and partly to help conserve the scarce resources of Yale’s breast cancer archive. With 236 patients in the training cohort and approximately 20% missing values, we decided to use multiple imputation on subsets of no more than 9 of the 42 markers. Figure 1 outlines the variable selection procedure; note that we are not concerned with model coefficients or prediction at this point.

We proceeded by conducting 1000 random allocations of the 42 markers to 5 groups of size 8, 8, 8, 9, and 9. For each of the resulting 5000 subsets of markers (with each marker appearing in 1000 subsets), multiple imputation was used to create 10 complete data sets. For each of these complete data sets, we fit a Cox proportional hazard model using a backwards stepwise variable selection procedure and the AIC penalty, collecting the t-statistics of the retained markers from each of the resulting subset models (as well as noting which variables were retained and which were eliminated by the stepwise procedure). Thus, each marker was studied in 10,000 subset models (with 1000 different random subsets of markers and 10 multiply imputed data sets for each subset). This use of random subsets of variables and the subsequent stepwise variable selection helps explore the high-dimensional space of models and focus attention on those markers of greatest prognostic value. We examined the mean t-statistics for coefficients of markers retained by the repeated stepwise procedures as well as the proportion of times each marker was retained. We then chose 15 of 42 markers for further study on the validation cohort. This procedure using multiple imputation is computationally intensive (taking approximately 2 hours in our study) compared to single-imputation methods (taking approximately 10 minutes).

### Statistical analysis: model selection

A secondary analysis of the training cohort identified a single, hypothesized “best” model containing baseline variables (age, tumor size, nuclear grade) and a smaller subset of markers. Again using multiple imputation, we used a procedure similar to forward stepwise selection to build the model. Variables were added one by one, each time by selecting that variable contributing the most to improving the log-likelihood (averaged over 100 multiply imputed data sets) and avoiding the inclusion of redundant markers. Our goal (guided by the biomedical research aims) was to identify a model containing not more than four to five markers; we examined improvements in the log-likelihood and Harrell’s R^2^ (Harrell, 2002) in choosing between four or five markers.

### Statistical analysis: out-of-sample validation

**Figure 2:**
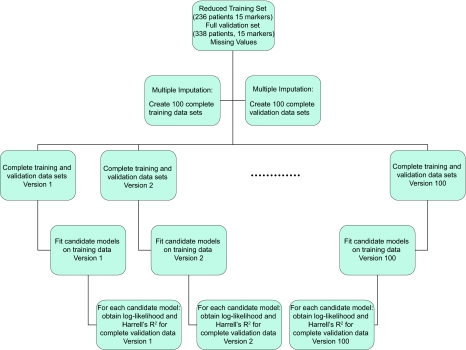
Out-of-sample validation.

Candidate models were identified and fit using only the training cohort; out-of-sample validation tests the goodness-of-fit and compares the models using the validation cohort. This provides an objective and rigorous means of validating the results of the study. Once again, missing values required specialized statistical analysis. Figure 2 outlines the validation procedure used to compare three models to a baseline clinical model including age at diagnosis, nuclear grade, and tumor size. The first model included positive nodes, the second included the four selected markers, and the full model added both positive nodes and the protein markers to the baseline model.

Multiple imputation was used to obtain 100 pairs of complete training and validation data sets. For each pair, candidate models were fit using the training cohort, and tested on the validation cohort. Goodness-of-fit statistics (the log-likelihood and Harrell’s R^2^) were obtained for each model on each of the imputed validation data sets. We compared the models by examining the distributions of the differences of the statistics between each of the three models of interest and the baseline model; p-values were calculated using the likelihood ratio test on the median improvement in the log-likelihood.

## Results

### Clinical and pathological variables

The training and validation cohorts included 236 and 338 patients, respectively, with histologically confirmed breast carcinoma. Ideally, the cohorts would have been selected completely at random, but this was infeasible given the limited tissue availability for some patients. The training cohort contained 110 events (46.6%), while the validation cohort contained 167 events (49.4%). Table 2 provides a comparison of the cohorts with respect to age at diagnosis, nuclear grade, tumor size and positive nodes using a univariate Cox proportional hazard analysis of survival. The cohorts were generally similar, although some differences were observed with respect to nuclear grade.

### Marker and model selection

Table 1 indicates the 15 markers (marked with an asterisk) selected for exploration in the validation cohort. In contrast, casewise deletion within the same randomly assigned subsets of variables followed by stepwise variable selection yielded a somewhat different set of recommended markers.

A baseline model was constructed using only clinical and pathological characteristics that would be available without axillary lymph node dissection. The model selection procedure examined the improvements from the baseline in the training cohort when adding nodal status and/or multiplexed biomarker protein expression level data. Analysis of the training cohort and consultations with the medical researchers resulted in a hypothesized “best” model, including COX6C, GATA3, NAT1, and ESR1. The addition of another marker provided negligible improvements in the log-likelihood and Harrell’s R^2^. Table 3 presents goodness-of-fit comparisons of several models including various combinations of markers and nodal status. We note that the study of the training set indicated that the markers might prove more useful than nodal status (“M4” provides significantly better improvements in goodness-of-fit than “Nodes” in Table 3), but that the best model includes both markers and nodal status.

### Out-of-sample validation

**Figure 3:**
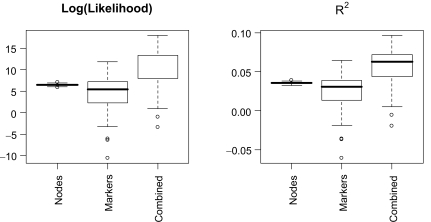
Validation of four-marker model Each plot depicts the distribution of improvements in the goodness-of-fit statistics for three candidate models compared to the baseline model containing only the clinical factors: “Nodes” (lymph node status and clinical factors); “Markers” (four selected protein markers and clinical factors), and “Combined” (including clinical factors, protein markers, and nodal status).

We validated the proposed four-marker model using the larger cohort of 338 patients. Figure 3 shows the distribution of differences in the goodness-of-fit statistics between the three candidate models and the baseline model. There were only a few missing values for nodal status (compared to 20% of the marker values), so we see less variability in the distribution of the statistics corresponding to the node model. The addition of these four markers provides significant improvements in the goodness-of-fit. The out-of-sample validation results differ somewhat from the study of the training set; we had expected markers to be more valuable than nodal status. The out-of-sample validation shows that the addition of either nodal status or the four protein expression levels provides similar (and significant) improvement over the baseline model (p-value < 0.001, and 0.028, respectively), while a full model using all available information provides the best improvement. The combined model provides a significantly better fit than the marker model (p-value < 0.001) or the nodal model (p-value 0.043). The distributions of the goodness-of-fit statistics resulted from the analysis of multiply imputed data sets, reflecting the uncertainty attributed to the missing protein expression measurements.

### Simulation

Investigators often drop cases with missing values from their studies. Other times, they may impute missing values using the mean or median values of each variable. A more sophisticated approach would use the k-nearest neighbor procedure for the purpose of imputation. Our approach, based on multiple imputation, offers an attractive alternative.

To demonstrate the advantages of our procedure, we generate 42 variables using the multivariate normal distribution with mean and covariance determined by the study data (and missing values assigned at random). We create a model for patient survival using the Weibull distribution and four coefficients for markers of decreasing levels of significance (taking values 4, 2, 1, and 0.5). The remaining 38 coefficients are set to 0, and we repeatedly simulate patient survival using this model. We would like a procedure to identify the helpful markers and yet not mistakenly select unimportant markers. For each of 50 simulated data sets, we apply four stepwise variable selection procedures to randomly selected blocks of data: our procedure, dropping cases with missing value, imputing with variable medians, and imputing using k-nearest neighbor value. To enable a fair comparison of these methods, each of the procedures selects variables based on t-statistics above 2 in absolute value.

Table 4 presents the results, showing, in particular, the danger of conducting an analysis by simply dropping cases having missing values. As expected, this is the worst alternative, rarely succeeding in identifying important variables. Imputing values using variable medians or k-nearest neighbor values does the best in terms of including important markers, but these methods also include a huge number of unimportant markets; they fail to account for the uncertainty due to the imputation of values, acting as if they have a complete data set. In contrast, our procedure based on multiple imputation does almost as well at selecting the important variables (having trouble only with the least significant of the four markers), but avoids including excessive numbers of irrelevant variables (only about 3 per attempt, compared to 5–6 for the other procedures).

## Discussion

This study examines the relative merits of using protein expression levels, invasive lymph node sampling and conventional clinical factors in breast cancer survival prognostic models. Automated quantitative analysis (AQUA™) was used to measure the protein expression levels of 42 markers on a breast cancer training cohort of 236 cases. We identify a small subset of markers (including COX6C, GATA3, NAT1, and ESR1) important in predicting patient survival, resulting in a model capable of predicting patient outcomes as effectively as a model utilizing nodal status alone. We validate the results on an independent cohort of 338 cases, finding that the addition of either nodal status or the four protein expression levels provides similar (and significant) improvement over a baseline model, while a full model using all available information provides the best patient predictions.

The availability of extensive tissue repositories coupled with annotated clinical information provide an opportunity to take advantage of the rich source of biologically relevant information in tissue specimens. Tissue microarrays provide a valuable resource for combining pathological, clinical, and biological data to develop predictive models for diseases such as cancer. Unfortunately, missing values are unavoidable in tissue microarray data for a variety of reasons. The tissue core might not contain tumor epithelium, cores might be missing, or the cores may be uninterpretable because of debris or other slide defects. In order to take full advantage of all available data, we incorporate multiple imputation with Cox proportional hazards modeling. This allows identification of a subset of markers of likely predictive value. Our simulation resulted provide one comparison of various methods of dealing with missing values; we note that an analysis of our training cohort that simply dropped cases with missing values would have overlooked the importance of two of the identified best markers.

It is not surprising that ESR1 (estrogen receptor alpha) is identified as an important component of the model since hormonal status was not otherwise used in the selection of the cohort. The biological relations that provide the additional value of these markers in the four marker model are not well studied, but deserve further exploration. Previous studies have shown that NAT1 expression is increased in breast tumors compared to normal breast tissue (Stanley, 1996). Bièche at al (2004) provide evidence that NAT1 may be an ERα-responsive gene in human breast cancer. NAT1 mRNA status (which has been shown to correlate well with immunohistochemistry for the NAT1 gene product) in this study also provided evidence of effect on prognosis independent of lymph node status. The mRNA levels of the transcription factor GATA3 and the Cytochrome c oxidase subunit Vic (COX6C), have both been reported to be important in discriminating hormone responsive breast cancer or with the ER+ subtype of tumors (for example, Gruvberger, 2001; Perou, 2000; West, 2001; Hoch, 1999; Pusztai, 2003). Mehra et al. (2005) recently reported that low expression of the transcription factor GATA3 was commonly present in invasive carcinomas with poor clinical outcome; its association with outcome has been reported by others (van de Rijn, 2002), along with its potential role in predicting hormonal therapy response (Parikh, 2005). Our results provide further evidence of the importance of low levels of this marker in poor risk breast cancer.

In summary, we propose a methodology for marker selection from a large number of biomarkers with missing data, and apply the methodology to biomarker discovery using breast cancer tissue microarrays. We found that a model including GATA3, COX6C, NAT1, and ESR1 provides equivalent prognostic value as lymph node status alone, and provides further information when combined with lymph node status. These results are strongly supported by out-of-sample model validation, and our methodology may be easily applied to other problems with missing data. The ability to conduct analyses in the presence of missing data will become increasingly important as tissue microarrays are used in research studies of drug responsiveness and clinically for patient prognosis.

## Figures and Tables

**Table 1:** Primary antibody details. *denotes those variables selected for analysis with the training cohort.

Antibody	Source	Species (Dilution, time)
ACADSB	Gift of Gerry Vockley {He, 2003}	rabbit polyclonal (1:5000, 1 hour)
AGR2	Gift of Devon Thompson {Thompson, 1998}	rabbit polyclonal (1:1000, 1 hour)
BCL2	DAKO, clone 124	mouse monoclonal (1:40, 1 hour)
BNIP3	BD Pharmingen	rabbit polyclonal (1:500, overnight)
CA12*	Gift of William Sly {Wykoff, 2001}	rabbit polyclonal (1:2000, 30 minutes)
CAV1*	Transduction Labs, clone 2297	mouse monoclonal (1:100, overnight)
CD24	Neomarkers, clone 24C02 Ab-2	mouse monoclonal (1:50, 1 hour)
CDH3*	BD Transduction Labs clone 56	mouse monoclonal (1:200, overnight)
COX6C*	Molecular Probes clone 3G5	mouse monoclonal (1:100, overnight)
CTSD	DAKO	rabbit polyclonal (1:1000, 1 hour)
EEF1D	Gift of Ong Lee Lee {Ong, 2003}	rabbit polyclonal (1:5000, 1 hour)
ESR1*	DAKO Estrogen Receptor antibody clone 1D5	mouse monoclonal (1:50, 1 hour)
GATA3*	Santa Cruz, clone HG3-31	mouse monoclonal (1:100, 1 hour)
GGH	Gift of Thomas J. Ryan {Rhee, 1998}	rabbit polyclonal (1:400, 1 hour)
GLUL	BD Transduction Labs clone 6	mouse monoclonal (1:1000, overnight)
GRB7	Santa Cruz	rabbit polyclonal (1:250, 1 hour)
GSTP1*	DAKO clone 353-10	mouse monoclonal (1:50, 1 hour)
HER2	DAKO	rabbit polyclonal (1:8000, 1 hour)
HSP27	Neomarkers clone Ab-1 G3.1	mouse monoclonal (1:50, 30 minutes)
IGFBP2	Santa Cruz	goat polyclonal (1:1000, 30 minutes)
IGFBP4*	Austral Biologicals	mouse monoclonal (1:50, 1 hour)
IGFBP5*	Austral Biologicals	mouse monoclonal (1:100, 1 hour)
IRAK1	Santa Cruz	rabbit polyclonal (1:100, 1 hour)
JUP	BD Transduction Labs	mouse monoclonal (1:1000, overnight)
KRT7	DAKO clone TL 12/30	mouse monoclonal (1:50, 1 hour)
KRT8	DAKO clone 25BH11	mouse monoclonal (1:100, 1 hour)
KRT18	DAKO clone DC10	mouse monoclonal (1:50, 1 hour)
KRT19	DAKO clone RCK108	mouse monoclonal (1:50, 1 hour)
MUC1	Novocastra	mouse monoclonal (1:100, overnight)
MYC*	DAKO clone 1D5	mouse monoclonal (1:200, 1 hour)
NAT1*	Gift of Edith Sim {Stanley, 1996}	rabbit polyclonal (1:1000, 1 hour)
PCNT1	Gift of Stephen Doxsey {Doxsey, 1994}	rabbit polyclonal (1:500, 1 hour)
PFK	Gift from George Dunaway {Dunaway, 1988}	rabbit polyclonal (1:2000, 1 hour)
RNF110	Santa Cruz	rabbit polyclonal (1:400, overnight)
SERPINA3	DAKO	rabbit polyclonal (1:3200, 10 minutes)
SLC7A5	Serotec	rabbit polyclonal (1:50, 1 hour)
SLC9A3R1	Gift of Vijaya Ramesh {Stemmer-Rachamimov, 2001}	rabbit polyclonal (1:50, overnight)
TFF1	DAKO clone BC04	mouse monoclonal (1:5000, overnight)
TFF3	Gift of Daniel Podolsky {Suemori, 1991}	rabbit polyclonal (1:500, 1 hour)
THBS1	Neomarkers clone A6.1 Ab4	mouse monoclonal (1:50, overnight)
TIMP3*	Oncogene Research clone 136-13H4 Ab-1	mouse monoclonal (1:50, overnight)
XBP1*	Santa Cruz	rabbit polyclonal (1:200, overnight)

**Table 2:** A comparison of the training and validation cohorts. Univariate Cox proportional hazard coefficients (with 95% confidence intervals) show the similarities between the cohorts with the exception of nuclear grade (which appears to have a statistically significant relationship to survival in the validation cohort, but not the training cohort).

Variable	Training (236)	Validation (338)
**Age at Diagnosis**		
Missing values (percent)	0 (0%)	0 (0%)
Mean (standard deviation)	59.9 (12.4)	56.8 (12.0)
Hazard ratio (95% confidence interval)	1.00 (0.989–1.02)	1.00 (0.987–1.01)
**Nodal Status and Positive Nodes**		
Missing values (percent)	1 (0.4%)	0 (0%)
Node positive (percent)	119 (50.4%)	169 (50%)
Node negative (percent)	116 (49.2%)	169 (50%)
Positive nodes: Mean (standard deviation)	6.6 (8.0)	6.0 (6.1)
Hazard ratio (95% confidence interval)	1.04 (1.02–1.07)	1.06 (1.04–1.07)
**Nuclear Grade**		
Missing values (percent)	9 (3.8%)	28 (8.3%)
1	36 (15.3%)	64 (18.9%)
Count (percent) 2	118 (50%)	169 (50%)
3	73 (30.9%)	77 (22.8%)
Hazard ratio (95% confidence interval)	1.12 (0.846–1.49)	1.39 (1.1–1.77)
**Tumor Size**		
Missing values (percent)	0 (0%)	37 (10.9%)
Mean (standard deviation)	2.97 (2.24)	Mean = 2.79 (2.12)
Hazard ratio (95% confidence interval)	1.10 (1.05–1.16)	1.15 (1.08–1.23)

**Table 3:** Model selection on training data.

Model name	Variables	Mean (standard deviation) Improvement over Baseline Clinical Model	
		R^2^	Log-likelihood
Baseline	Age at Diagnosis Nuclear Grade Tumor Size	NA	NA
M1	Baseline + COX6C	0.0537 (0.0157)	6.78 (2.05)
M2	M1 + GATA3 (N)	0.0655 (0.0158)	8.33 (2.08)
M3	M2 + ESR1 (N)	0.0693 (0.0155)	8.82 (2.06)
M4	M3 + NAT1.Total	0.0743 (0.0170)	9.48 (2.26)
Nodes	Baseline + Positive Nodes	0.0312 (0.0011)	3.89 (0.13)
Combined	Baseline + M4 + Positive Nodes	0.0993 (0.0171)	12.85 (2.35)

**Table 4:** Simulation results. The table shows the number of times (out of 50) that the four markers were captured by the variable selection process. The last column indicates the mistaken inclusions of spurious variables.

Method	Beta1	Beta2	Beta3	Beta4	Others
stepMI	50	50	50	6	159
Drop	9	4	2	0	35
Median	50	50	50	43	572
KNN	50	50	50	37	512
